# Patterns of gene expression associated with recovery and injury in heat-stressed rats

**DOI:** 10.1186/1471-2164-15-1058

**Published:** 2014-12-03

**Authors:** Jonathan D Stallings, Danielle L Ippolito, Vineet Rakesh, Christine E Baer, William E Dennis, Bryan G Helwig, David A Jackson, Lisa R Leon, John A Lewis, Jaques Reifman

**Affiliations:** Environmental Health Program, U.S. Army Center for Environmental Health Research, Bldg. 568 Doughten Drive, MD 21702-5010 Fort Detrick, Maryland; Oak Ridge Institute for Science and Education, Fort Detrick, Maryland; DoD Biotechnology High Performance Computing Software Applications Institute, Telemedicine and Advanced Technology Research Center, U.S. Army Medical Research and Materiel Command, Fort Detrick, Maryland; Excet, Inc., Fort Detrick, Maryland; Thermal Mountain Medicine Division, U.S. Army Research Institute of Environmental Medicine, Natick, Massachusetts; Pulmonary Health Program, U.S. Army Center for Environmental Health Research, Fort Detrick, Maryland

**Keywords:** Heat stress, Transcriptomics, Proteomics, Systems biology, Protein aggregation

## Abstract

**Background:**

The *in vivo* gene response associated with hyperthermia is poorly understood. Here, we perform a global, multiorgan characterization of the gene response to heat stress using an *in vivo* conscious rat model.

**Results:**

We heated rats until implanted thermal probes indicated a maximal core temperature of 41.8°C (T_c,Max_). We then compared transcriptomic profiles of liver, lung, kidney, and heart tissues harvested from groups of experimental animals at T_c,Max_, 24 hours, and 48 hours after heat stress to time-matched controls kept at an ambient temperature. Cardiac histopathology at 48 hours supported persistent cardiac injury in three out of six animals. Microarray analysis identified 78 differentially expressed genes common to all four organs at T_c,Max_. Self-organizing maps identified gene-specific signatures corresponding to protein-folding disorders in heat-stressed rats with histopathological evidence of cardiac injury at 48 hours. Quantitative proteomics analysis by iTRAQ (isobaric tag for relative and absolute quantitation) demonstrated that differential protein expression most closely matched the transcriptomic profile in heat-injured animals at 48 hours. Calculation of protein supersaturation scores supported an increased propensity of proteins to aggregate for proteins that were found to be changing in abundance at 24 hours and in animals with cardiac injury at 48 hours, suggesting a mechanistic association between protein misfolding and the heat-stress response.

**Conclusions:**

Pathway analyses at both the transcript and protein levels supported catastrophic deficits in energetics and cellular metabolism and activation of the unfolded protein response in heat-stressed rats with histopathological evidence of persistent heat injury, providing the basis for a systems-level physiological model of heat illness and recovery.

**Electronic supplementary material:**

The online version of this article (doi:10.1186/1471-2164-15-1058) contains supplementary material, which is available to authorized users.

## Background

Heat illness, a continuum of disorders caused by hyperthermia, includes heat cramps, heat exhaustion, heat injury, and heat stroke [1–3]. Hyperthermia occurs when the body’s core temperature exceeds its hypothalamic set point as a result of overwhelming external or internal sources of heat or the effects of drugs or disease which impair heat dissipation [1–3]. Without adequate fluid replacement, dehydration and loss of blood volume lead to circulatory collapse, severe hyperthermia, and eventually heat stroke, defined as neurological dysfunction associated with a body temperature higher than 40°C (104°F) in humans [3–5].

Sudden death related to heat stroke occurs primarily in individuals with pre-existing cardiac abnormalities, organ failure secondary to rhabdomyolysis, or acute onset of organ failure [6]. Systemic inflammatory response syndrome (SIRS) is considered to be the primary cause of organ dysfunction related to heat stroke [3, 7, 8], resulting in severe encephalopathy, rhabdomyolysis, acute renal failure, acute respiratory distress syndrome, myocardial injury, hepatocellular injury, intestinal ischemia, pancreatic injury, and hemorrhagic complications [3]. SIRS may also be complicated by endotoxemia and/or disseminated intravascular coagulation through leakage of the gut epithelium [8] and/or damage to the vascular endothelium [9], respectively (particularly exertional heat stroke). Thus, multiorgan dysfunction syndrome is the primary reason for heat-related morbidity and mortality.

At the cellular and molecular levels, severe hyperthermia causes acidosis, hypoxia, and cellular fatigue. Thermal stress leads to an increased expression of heat shock proteins (HSPs) associated with thermotolerance [10–13]. The cellular response extends beyond HSPs to include networks of gene changes in organs and peripheral blood mononuclear cells after whole-body hyperthermia [14] or heat stroke [15]. Moran and colleagues [10] describe at least three components of a time-dependent cascade of events related to the heat-stress response: *1*) the involvement of HSPs, *2*) a response that involves interferon-inducible genes (e.g., cytokines), and *3*) a small, nonspecific stress response shared by other cell lines and stressors [11]. Heat stress–induced molecular perturbations are also a likely contributing factor [10, 15–19] to altered heat intolerance [11].

Recently, we described an anatomically accurate, three-dimensional rat model of thermoregulation during heat stress [20], in which rats were placed at 37°C until their core temperature reached 41.8°C (T_c,Max_); they were then allowed to recover. Here, we perform a global characterization of the heat-stress–induced gene response in liver, lung, kidney, and heart tissues at T_c,Max_, and during recovery at 24 hours and 48 hours. Previous studies examined transcriptional alterations related to heat stress in various mammalian models and tissues [10, 11, 15, 17–19, 21, 22]. In this study, we use a whole transcript, gene array approach, which avoids issues related to 3’ bias, genes which lack poly-A tails, and confounders related to isoform variation [23]. We present a pathway analysis to establish the regulatory framework of heat stress in our conscious rat model. Within this framework, we examine the consensus heat-stress response (cHSR) found in all four organs, and discuss genes not previously linked to *in vivo* heat stress. Finally, we identify changes in gene and protein expression in the heart which are anchored to histological evidence of organ injury, and we propose that mitochondrial dysfunction and injury pathways are involved in the observed cardiomyopathy.

## Results and discussion

**Figure 1:**
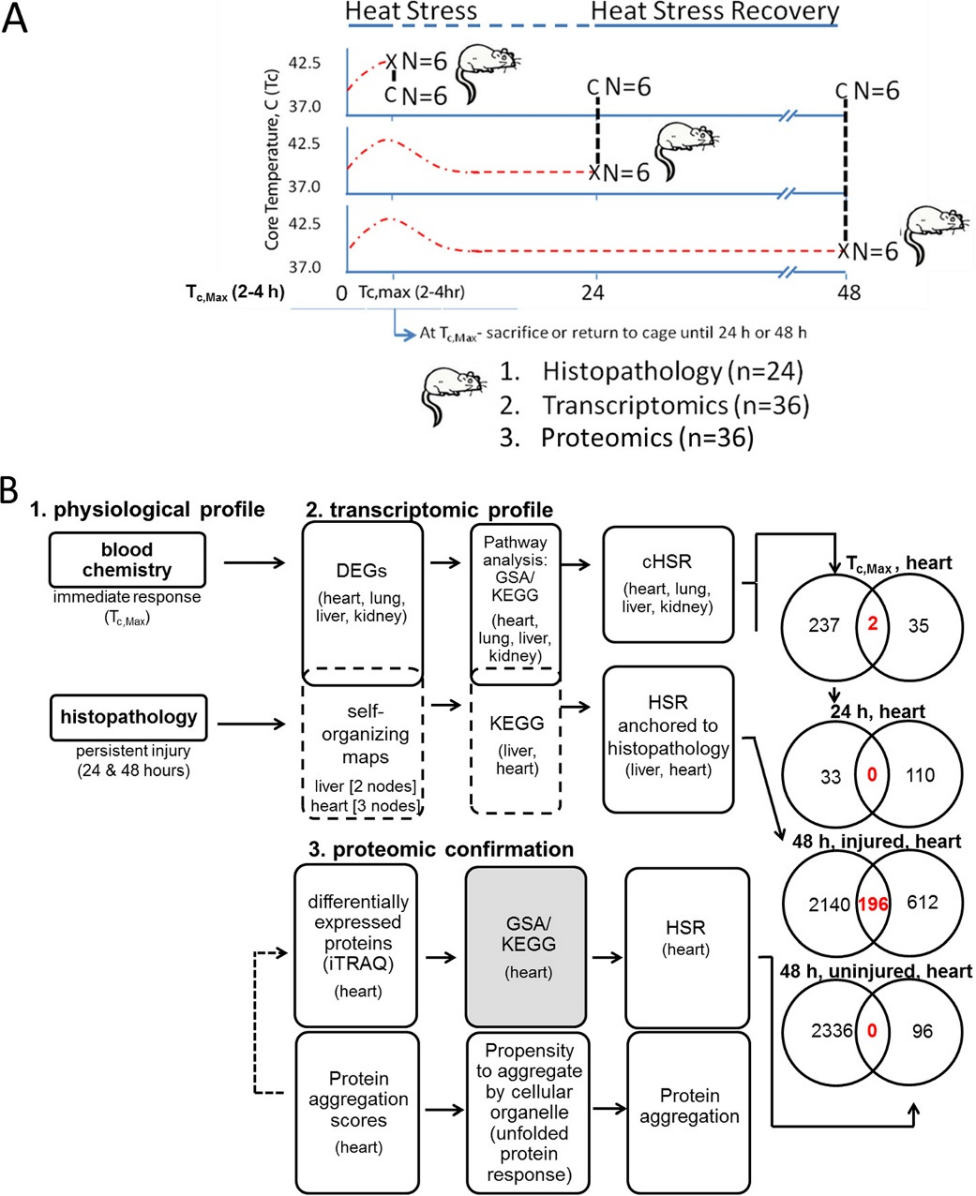
**Heat stress experimental design. (A)** Core temperature (Tc) was monitored by telemetry probe in real time in heated rats [X] or time-matched controls [C], and animals were sacrificed at Tc,Max (41.8°C), 24 hours, or 48 hours. Tissues were preserved for histopathology, transcriptomics, and proteomics. **(B)** Blood chemistries and histopathology were evaluated. Differential gene expression, gene set analysis, and KEGG enrichment were examined in all organs at Tc,Max to characterize the consensus gene response to heat stress. Self-organizing maps were constructed to identify genes that clustered in individual responders. We evaluated proteomic changes in the heart at Tc,Max, 24 hours, and 48 hours. Animals in the 48-hour cohort were subdivided by histopathology (injured and uninjured). Venn diagrams indicate the numbers of genes (left) and proteins (right) identified in each respective analysis. Red text denotes statistically significant changes in the same genes and proteins.

To induce the pathophysiological effects of heat stress, we placed conscious rats in an incubator at 37°C and monitored their core temperatures using remote telemetry implants (Figure 1A; see also Rakesh *et al.* [20]). Animals were euthanized when their core temperature reached 41.8°C (T_c,Max_), or they were returned to their normal housing environment and allowed to recover for up to 48 hours prior to sacrifice. We characterized the response of the animals to heat stress using a standard panel of hematological and clinical chemistry assays, as well as a histopathological evaluation of the liver, heart, kidney, and lung. In the context of these well-established assays, we also analyzed global changes in gene expression in the four tissues using microarrays and performed a “shotgun” proteomic analysis of cardiac tissue using mass spectrometry. This approach allowed us to link changes in gene and protein expression to tissue injury, the animals’ physiological state, and their overall health (Figure 1B).

### Physiological profiles

Complete blood count and concomitant decrease in body weight were significantly different between heat-stressed animals and time-matched control animals only at T_c,Max_ (Additional file 1: Table S1). Further, blood urea nitrogen (BUN), a measure of kidney function, was significantly higher in heat-stressed animals, consistent with dehydration and/or kidney function impairment (Additional file 1: Table S1). As expected, no animals demonstrated significant heat-related histopathological changes at T_c,Max_ (Additional file 2: Table S2). The incidence and severity of lesions, such as cardiomyopathy and chronic progressive nephropathy, were generally minimal and consistent with the normal background lesions found in rats at 2–3 months of age (Additional file 2: Table S2).

Animals sacrificed at 24 hours demonstrated background lesions which were minimal in severity and not considered related to heat exposure (Additional file 3: Table S3). In one animal, we found diffuse, acute tubular necrosis in the kidney (Additional file 4, Figure S1A, B) and acute necrosis in the liver, mostly affecting hepatocytes in the periportal regions (Additional file 4: Figure S1C, D). Similar lesions did not occur in any other animal at any other sacrifice time point.

**Figure 2:**
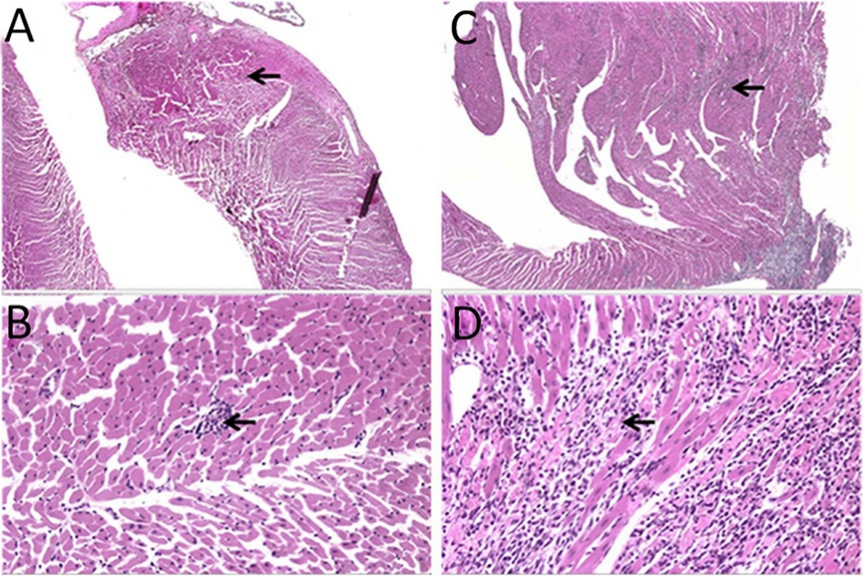
**Histopathological evidence of cardiomyopathy and inflammation around cardiomyofibers at 48 hours in heat**-**stressed rats** (**hematoxylin and eosin staining).** Minimal cardiomyopathy in unheated control rats is observed at **(A)** 20× and **(B)** 200× magnification. Arrows represent focal areas of cardiomyopathy with few macrophages and rare neutrophils. **(C)** Cardiac sections from heat-stressed animals show moderate inflammatory infiltrate (arrows) at 48 hours (20× magnification). **(D)** Inflammation separates and surrounds the cardiomyofibers with occasional degenerative cardiomyofibers (200× magnification).

Although we found essentially no evidence of physiological or histopathological changes related to heat stress at 24 hours, we observed substantial cardiac injury in three out of the six animals sacrificed at 48 hours (Additional file 5: Table S4). All treated animals (and one control) had degeneration of cardiomyocytes with subacute inflammation consisting of mostly macrophages. This type of lesion is common in Fisher 344 rats (Figure 2A and B, time-matched controls), but the pattern of injury and severity of the degeneration and inflammation observed at the 48-hour time point suggested treatment-related tissue injury rather than an incidental, strain-related cardiomyopathy (Figures 2C and D). Extensive tissue damage often leads to kidney proteinosis similar to that observed in the animals most severely affected by cardiac inflammation (Additional file 5: Table S4). Thus, while the physiological parameters at T_c,Max_ suggest that the animals had undergone heat-related dehydration, the development of tissue injury appeared to be a delayed process. No injury was evident at 24 hours, but there was clear, persistent, heat-induced cardiac damage in some animals at 48 hours.

### The consensus heat-stress response: pathway analysis of the transcriptome at T_c,Max_

In order to elucidate the biological events related to heat stress at the molecular level, after heat exposure we identified genes which were differentially expressed in the heart, lung, liver, and kidney using Partek® Genomics Suite software (version 6.3 Copyright © 2008 Partek Inc., St. Louis, MO, US) (Additional file 6: Table S5), and we performed a functional enrichment analysis of the genes using the Database for Annotation, Visualization, and Integrated Discovery (DAVID) [24, 25]. Gene set analyses were conducted in Partek to determine the top enriched gene ontology (GO) terms for each condition (Additional file 7: Table S6). At T_c,Max_, 467 unique biological processes (BPs), cellular components (CCs), and molecular functions (MFs) were enriched across all organs, of which only 25 were common to all four organs, and 156 were common at least to two organs. Using DAVID, we also determined enriched Kyoto Encyclopedia of Genes and Genomes (KEGG) pathways (Additional file 8: Table S7). The differentially expressed genes (DEGs) from all four organs were enriched for functional terms related to protein folding and/or response to unfolded protein, regulation of apoptotic process, and the response to lipopolysaccharide (LPS)/cytokine in the gene set enrichment analysis. There was also a set of stress and transcriptional responses associated with the mitogen-associated protein kinase (MAPK) KEGG pathway (Table 1).

**Table 1:** **Gene set and KEGG enrichment analysis of the consensus heat-stress response at T**_**c,Max**_

Function ^*^	Transcript ID	Type ^*^	Heart ES	Liver ES	Kidney ES	Lung ES
**Protein folding/unfolding response**						
Protein folding	6457	BP	23.5	10.9	13.3	39.8
Unfolded protein binding	51082	MF	14.4	12.0	16.0	32.7
Heat-shock protein binding	31072	MF	13.4	5.8	7.3	15.9
Response to heat	9408	BP	9.3	15.2	10.7	13.6
Chaperone binding	51087	MF	7.6	8.6	12.7	8.5
Response to unfolded protein	6986	BP	7.3	14.9	19.9	19.1
**Response to apoptosis**						
Positive regulation of apoptotic process	43065	BP	11.8	12.3	9.3	12.5
Negative regulation of neuron apoptotic process	43524	BP	7.9	8.3	7.5	7.7
**Response to LPS/cytokines**						
Response to lipopolysaccharide	32496	BP	5.7	10.1	3.2	11.1
Response to cytokine stimulus	34097	BP	4.8	6.2	4.5	15.5
**Transcriptional Response/Other**						
RNA polymerase II core promoter proximal region sequence-specific DNA binding transcription factor activity involved in positive regulation of transcription	1077	MF	15.4	6.6	10.5	6.1
Response to cAMP	51591	BP	10.7	3.1	5.5	4.8
Double-stranded DNA binding	3690	MF	8.4	7.0	8.5	7.8
Response to mechanical stimulus	9612	BP	8.3	5.6	5.1	4.6
Response to hydrogen peroxide	42542	BP	7.9	6.6	8.1	5.9
Transcription factor complex	5667	CC	7.5	4.8	5.6	5.0
Positive regulation of smooth muscle cell proliferation	48661	BP	6.2	3.6	6.1	4.9
Response to glucocorticoid stimulus	51384	BP	5.8	11.5	4.8	3.9
Negative regulation of transcription from RNA polymerase II promoter	122	BP	5.8	6.0	7.0	4.8
Response to hypoxia	1666	BP	5.5	8.4	7.8	12.3
Response to estrogen stimulus	43627	BP	5.5	5.9	8.1	4.7
Cell surface	9986	CC	5.1	7.0	4.8	6.7
Positive regulation of angiogenesis	45766	BP	4.8	6.6	8.7	10.8
Response to light stimulus	9416	BP	3.8	7.6	10.4	6.0
Response to drug	42493	BP	3.7	7.8	6.9	3.2
**MAPK Pathway**	rno04010	KEGG	5.0	2.8	5.3	3.3

^*^GO terms significantly enriched in liver, kidney, heart, and lung (25 common to all organs out of 467 enriched terms at T_c,Max_). BP, biological processes; cAMP, cyclic adenosine monophosphate; CC, cellular components; ES, enrichment score; KEGG, Kyoto Encyclopedia of Genes and Genomes pathway; LPS, lipopolysaccharide; MAPK, mitogen-associated protein kinase; MF, molecular functions; T_c,Max_, maximum core temperature.

At T_c,Max_, we identified 78 differentially expressed transcripts in all four organs (Additional file 6: Table S5), comprising a cHSR. We examined the cHSR further with DAVID tools and found that 51 nonredundant transcript identifiers (IDs) were identified in 11 functional annotation clusters (Additional file 9: Table S8). The most significantly enriched terms in the dataset were then subcategorized as “protein folding” (PF) and “regulation of apoptosis” (RA) (Table 2). Twenty-seven transcripts were not functionally clustered by DAVID (Additional file 6: Table S5), but literature supports the roles related to PF [26–34] and/or RA [35, 36] in 14 of the 27 (Additional file 6: Table S5). The remaining 13 transcripts had unknown functions or were related to inflammation or other biological processes [37–41] (Additional file 6: Table S5).

**Table 2:** **Consensus genes functionally clustered to “protein folding” and “regulation of apoptosis” at T**_c,Max_^*^

			Fold change from control (unheated)			
Transcript iD	Gene symbol	Functional process	Heart	Liver	Kidney	Lung
0810295	*Dnajb1*	PF	24.1	17.1	9.7	10.5
10756343	*Hsph1*	PF	14.8	12.4	11.6	15.7
10819787	*Dnajb4*	PF	4.5	6.3	3.5	3.7
10923050	*LOC501135*	PF	3.1	3.4	2.8	2.0
10939480	*Morc4*	PF	2.4	2.3	2.1	2.7
10869047	*Hspa8*	PF	2.2	3.4	2.1	2.9
10774470	*Hspa8*	PF	2.2	2.4	2.1	2.6
10927699	*LOC363224*	PF	2.1	2.9	2.7	3.1
10816048	*N5*	PF	2.1	2.3	2.1	2.6
10828154	*Hspa1b*	PF/RA	80.0	209.2	50.2	67.2
10886031	*Fos*	PF/RA	24.9	9.7	3.5	4.1
10827231	*Cyr61*	PF/RA	12.8	7.5	9.5	4.2
10806122	*Hmox1*	PF/RA	11.0	7.7	14.4	8.1
10878112	*Jun*	PF/RA	6.7	12.5	8.4	4.0
10868289	*Dnaja1*	PF/RA	6.7	2.6	3.6	5.5
10937318	*Hsp90aa1*	PF/RA	6.3	4.4	3.0	4.7
10892184	*Hsp90aa1*	PF/RA	6.0	6.5	5.9	5.0
10845384	*Nr4a2*	PF/RA	5.1	2.6	2.1	2.0
10723884	*Serpinh1*	PF/RA	3.4	4.3	6.0	3.4
10721865	*Ppp1r15a*	PF/RA	3.2	3.5	2.8	2.6
10910084	*Dnaja4*	PF/RA	2.9	25.2	4.7	4.7
10776667	*Hspd1*	PF/RA	2.6	3.1	2.3	6.1
10928154	*Hspd1*	PF/RA	2.3	3.6	2.4	6.9
10865855	*Fkbp4*	PF/RA	2.2	2.2	4.0	6.1
10895861	*Ddit3*	PF/RA	2.1	3.1	3.6	2.8
10770710	*Atf3*	RA	54.8	45.8	20.0	6.1
10868940	*Nr4a3*	RA	17.6	4.3	8.8	7.3
10761047	*Serpine1*	RA	12.3	2.4	4.8	6.0
10899387	*Nr4a1*	RA	10.5	6.0	4.1	2.4
10757082	*Zfand2a*	RA	8.1	27.8	7.7	6.9
10761128	*Hspb1*	RA	6.5	52.8	20.6	11.2
10711401	*Bag3*	RA	5.2	16.0	8.2	11.4
10931308	*P4ha1*	RA	5.0	7.0	5.5	4.4
10933015	*MGC95208*	RA	4.7	2.8	2.3	3.4
10769131	*Cacybp*	RA	4.6	3.6	3.1	6.7
10752650	*MGC95208*	RA	4.5	2.8	2.3	3.4
10940689	*Ifrd1*	RA	4.2	6.4	4.0	2.5
10917116	*Zbtb16*	RA	3.7	3.0	5.2	6.8
10749352	*Jmjd6*	RA	3.3	3.0	2.6	3.3
10732652	*Dusp1*	RA	2.9	2.1	3.5	4.2
10728361	*Stip1*	RA	2.9	3.7	3.7	5.7
10842469	*Cebpb*	RA	2.9	2.4	2.4	2.0
10940529	*Cebpd*	RA	2.7	2.5	2.9	3.4
10764643	*Trmt1l*	RA	2.5	3.2	2.2	3.1
10939516	*Tsc22d3*	RA	2.5	5.4	3.9	4.5
10702309	*Tbpl1*	RA	2.5	2.3	2.3	2.4
10758134	*Ubb*	RA	2.5	2.9	3.0	3.8
10794225	*Nfil3*	RA	2.3	2.8	4.2	2.6
10778763	*Rel*	RA	2.2	2.9	2.1	2.1
10861560	*N5*	RA	2.1	3.1	2.3	2.7
10795616	*Crem*	RA	2.0	2.4	2.5	2.9

^*^PF, protein folding; RA, regulation of apoptosis; T_c,Max_, maximum core temperature.

The unfolded protein response is activated in response to the accumulation of unfolded or misfolded proteins in the lumen of the endoplasmic reticulum (ER). Once activated, global protein translation is halted and chaperones (e.g., HSPs) are produced to manage protein folding. If the unfolded protein response fails, a cellular alarm response occurs, leading to autophagy and recovery [42–48] or apoptosis [49] and/or cytolysis [50]. At the molecular level, imbalance of the proteostasis network results in a cascade of responses to cellular stressors, involving molecular chaperones, clearance mechanisms, detoxifying enzymes, and an accumulation of misfolded aggregation-prone and proteotoxic species, which can lead to cell-specific dysfunction [50, 51]. The heat shock factor 1 (*Hsf1*) cycle can be initiated by proteotoxic stress, ultimately leading to the expression of heat shock proteins [10, 15], however, less is known about how heat stress, proteostasis networks, apoptosis, and autophagy contribute to organ-specific injury. In the heart, aberrant folding and unfolded protein response signaling lead to a number of pathologies, including myocardial ischemia, cardiac hypertrophy, and heart failure [52, 53]. Thus, the enriched pathways in protein folding and unfolded protein response at T_c,Max_ may point toward a global imbalance of the proteostasis network [50].

### The consensus heat stress response: differential gene expression in protein folding and regulation of apoptosis pathways at T_c,Max_

The environmentally induced heat shock transcripts and heat-stress–related transcripts enriched in PF and RA displayed the largest fold changes in heat-stressed animals relative to controls at T_c,Max_ (Table 2). Interestingly, our three-dimensional thermoregulatory model for the rat predicted that the liver temperature in heat-stressed animals exceeds that of other organs [20]. This prediction is consistent with our findings that the largest changes in temperature-dependent HSPs occurred in the liver.

A wide variety of physiological and environmental stimuli can induce expression of HSPs, including physical, chemical, and biological insults (e.g., inflammation) [54]. Genes encoding HSPs are divided into five superfamilies based on molecular weight: the small HSPs [55], the *Hsp40* (*DnaJ*) family, the *Hsp70* (*Hspa*) family, and the *Hsp90* (*HspC*) and *Hsp110* (*HspH*) families [56]. As molecular chaperones, HSPs play central roles in *1*) protein folding, aggregation, transport, and/or stabilization; *2*) cytoprotection, by preventing post-mitochondrial apoptosis in caspase-dependent pathways (e.g., *Hspb1*, *Hspa1b*, and *Hsp90*) and/or caspase-independent pathways (e.g., *Hsp70*) [57]; and *3*) cytoprotection, by preventing apoptosis at the premitochondrial level through inhibition of stress-activated kinases (i.e., *Jnk1*) in an adenosine-triphosphate (ATP)-independent manner (e.g., *Hsp70*) [58]. Although a recent report demonstrates a heat-stress–induced increase in rat liver expression of the inducible HSP70 protein (HSP72) but not the constitutive forms (HSP70 or HSP73) [59], our study demonstrates large increases in the products of both constitutive and inducible HSP genes at T_c,Max_ (Table 2), including *Hspb1*, *Hspa1b*, *Hspe1*, *Serpinh1*, *Hspd1*, *Hsp90aa1*, *Hsph1*, *and* several members of the *DnaJ* (Hsp40) chaperone family, transcripts homologous to genes coding for HSPs [i.e., *LOC498996* (similar to *DnaJ* homolog subfamily A, member 4), *LOC500476* (similar to *Hsp8*), and *RGD1561150* (similar to genes encoding CPN10-like protein)], and heat-stress–related genes (e.g., *Slc5a3*, *SNORD14*, *Ahsa2*, *Chordc1*, and *Banp*). *SNORD14* encodes a small nucleolar RNA which is transcribed from intronic regions of the *Hsp70* gene in mice [27]. Heat-stress–induced expression of *SNORD14* may facilitate the repair process involving ribosomal RNA after heat injury [27]. The environmentally inducible HSP gene, *Hspa1b,* was expressed at very high levels in all tissues (ranging from 24- to 209-fold), although one copy of the gene did not functionally cluster with the PF response in DAVID (Additional file 6: Table S5). *Hspa1b* codes for an ATP-dependent chaperone regulating intracellular anti-apoptosis, and is considered to be the most universal stress-induced HSP [54, 60, 61]. In baboons, expression of *Hsp72* is proportional to severity of heat stress [62].

Some of the differentially expressed HSPs at T_c,Max_ are also found in the extracellular environment (including *Hspb1* and *Hspa1b*), where they are thought to have immunogenic functions (Table 2) [54]. Putative mechanisms for HSP release into biofluids include cell death, active non-classical secretory pathways, lysosome-endosome pathways, release by secretory-like granules, or insertion into the lipid bilayers of export vesicles. The protein product of extracellular *Hsp72* binds to CD14, toll-like receptor (TLR) 4, and CD40 on dendritic cells, monocytes, and macrophages [63–65] and induces expression of NF-κB and chemokines (MIP-2 or CXCL2) by a TLR 2/4-dependent mechanism [66, 67]. It remains to be determined whether products of the differentially expressed HSP genes in this study are actually released into the extracellular space in our heat stress model.

The apoptosis pathway was also significantly enriched in the KEGG analysis. In the heart, liver, and kidney, activating transcription factor 3 (*Atf3*) was significantly induced (Table 2). The protein product of *Atf3* is a member of the ATF/cyclic AMP response element-binding (ATF/CREB) family of transcription factors [68, 69]. The product of *Atf3* is a critical negative regulator of pro-inflammatory cytokine production in response to TLR activation [70]. *Atf3* knockout mice exhibit no developmental abnormalities [71] unless challenged with LPS or another stress stimulus, resulting in an overt susceptibility to endotoxic shock and death [70]. *Zfand2a*, also known as arsenite-inducible RNA-associated protein (AIRAP), was also up-regulated in all four organs in heated animals (Table 2). In rats, *Zfand2a* is closely associated with the febrile response, a heat-associated physiology. Although early experiments in rodent fibroblasts suggested that *Zfand2a* was induced by arsenite stress but not heat shock, later experiments in human cell lines demonstrated that *Zfand2a* was induced by heat stress in an HSF-1–dependent manner and closely paralleled *Hsp70* expression after heat stress. While we can associate most of the members of the cHSR with heat stress, a few genes are associated with other stressors and some are uncharacterized (see Additional file 6: Table S5). Up-regulated apoptosis-related genes included *Uspl1*, *MGC95208*, and *Dusp8* [72, 73]. How the products of these genes contribute to the consensus response will require further investigation.

No transcripts were down-regulated in the cHSR, although several transcripts were down-regulated in at least three organs and were near the threshold for the fourth organ. For example, *Cd180* was down-regulated in the liver, kidney, and lung, but was just below the two-fold significance threshold in the heart (i.e., 1.99-fold down-regulated; see Additional file 6: Table S5). *Cd180* codes for a cell surface marker in the TLR family that forms the receptor RP105/MD1 and negatively regulates the expression of TLR4 [74, 75]. Reduction of the *Cd180* expression by heat stress may promote TRL signaling and inflammation.

### Transcriptomic profile at 24 and 48 hours

Our results at T_c,Max_ provide evidence of genes and functional regulatory pathways involved with the initial *in vivo* heat-stress response. Most of the DEGs at T_c,Max_ in the cHSR returned to control levels by 24 hours after heat stress (Additional file 6: Table S5). These results are consistent with the blood chemistries, suggesting that animals achieved recovery of most organ function by 24 hours. At T_c,Max_ and 24 hours, heat-stressed animals showed limited histopathological effects, although one animal showed evidence of liver histopathology at 24 hours (Additional file 3: Table S3 and Additional file 4: Figure S1). However, at 48 hours in three out of six animals, both histopathology and the pattern of gene expression indicated evidence of persistent, moderately severe damage to heart tissue.

**Figure 3:**
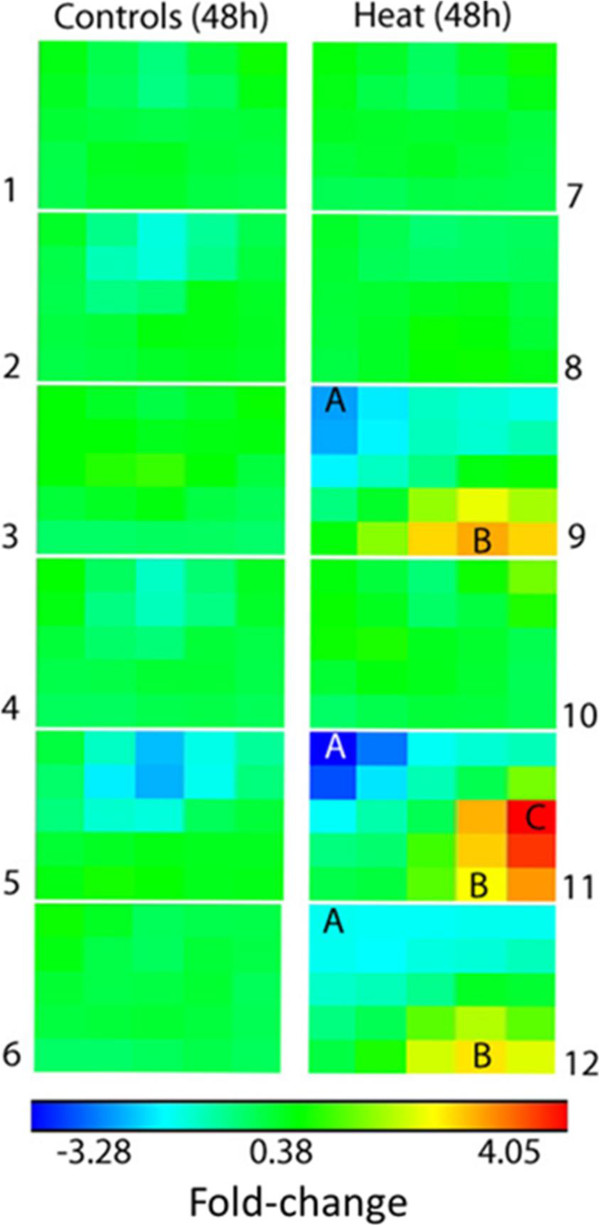
**Heart self-organizing map identifies genes that anchor to histopathological changes at 48 hours.** Node A (1351 down-regulated genes) and Node B (887 up-regulated genes) were common to the three animals (9, 11, and 12) with the most severe histopathological injury. See Additional file 8 for a full list of transcript IDs and the KEGG analysis of Nodes **A**, **B**, and **C**.

We explored the patterns of gene expression in our experimental animals using self-organizing maps, and observed that the DEGs in the hearts of three out of six heat-stressed animals at 48 hours differed from both controls and the other heat-stressed animals (Figure 3 and Additional file 8: Table S7; see Additional file 8: Table S7 and Additional file 10: Figure S2 for a self-organizing map analysis of the single animal with liver injury at 24 hours). Strikingly, these same animals also displayed cardioinflammation in the histological analysis. Node A (Figure 3) contains 1,351 down-regulated transcripts and Node B contains 887 up-regulated transcripts (see Additional file 8: Table S7). We next performed a functional analysis of the DEGs in Nodes A and B using DAVID, because these nodes were evident in the three animals with the highest histopathological scores. The down-regulated genes were associated with highly enriched KEGG pathways, including oxidative phosphorylation (enrichment score of 6.7, 63 genes), cardiac muscle contraction (enrichment score of 6.2, 33 genes), and pathways associated with chronic proteotoxic stress [Parkinson’s disease (enrichment score of 6.3 fold, 61 genes), Huntington’s disease (enrichment score of 4.8, 63 genes), and Alzheimer’s disease (enrichment score of 4.6, 62 genes)]. The oxidative phosphorylation pathway is central to Parkinson’s Disease, Huntington’s Disease, Alzheimer’s Disease, and Cardiac Muscle Contraction, suggesting that mitochondrial dysfunction and disruption in energetics are key features in animals with heat-stress-induced heart injury. Mitochondrial dysfunction, disruption of ER and membrane trafficking, alterations in protein folding and clearance, and activation of inflammation are all thought to be underlying causes of protein-folding disorders in neurons and other organ systems [76–79]. Primary and secondary aggregation of peptides and proteins disrupt normal protein homeostasis, leading to pathological states involving aberrant protein aggregation [76–79]. A recent study shows a strong correlation between cytological evidence of aggregated proteins and the functional pathways we observed in our clustering analysis (i.e., Oxidative Phosphorylation, Parkinson’s Disease, Huntington’s Disease, Alzheimer’s Disease, and cardiac muscle contraction [80]). Further, supersaturated protein complexes in protein aggregates are associated with highly enriched Ribosome and Proteasome pathways. The Ribosome pathway is enriched in genes upregulated in node C (+2.8 fold), cell cycle (+5.7 fold), and DNA replication (+6.3 fold) (Additional file 8: Table S7). Thus, the three animals with cardiac injury in response to heat stress show genomic signatures consistent with neurodegenerative diseases at the pathway level, suggesting that heat-injured animals have transcriptomic evidence of a greater propensity of protein to aggregate.

### Proteomic analysis of heart tissue at T_c,Max_, 24 hours, and 48 hours

**Figure 4:**
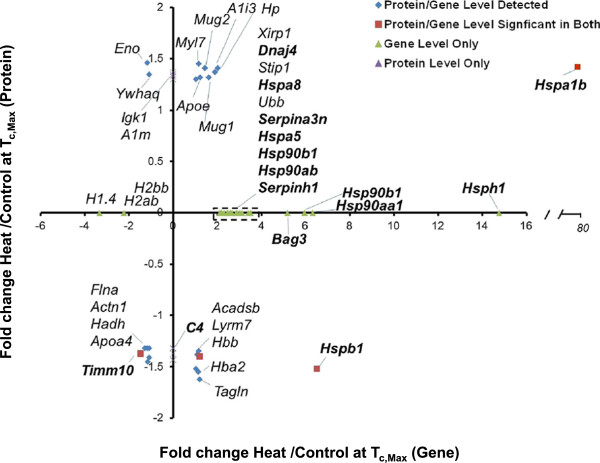
**Differentially expressed genes and proteins and KEGG pathway enrichment analysis in hearts at T**_**c,Max**_ **.** Proteins were mapped to gene probes to compare differentially expressed proteins and genes in the heart. Heat shock proteins and proteins related to HSPs are bolded. Proteins expressed at ±1.3-fold and their respective genes (±2-fold) are represented by red squares. Proteins and genes which mapped together but did not meet significance at the gene level are represented as blue diamonds. Genes (green triangles) or proteins (purple triangles) that were expressed and significant, but did not map together, are plotted along the axes. For clarity, only the gene designations are listed.

To determine whether the pathway analysis was complementary at the protein level, proteins were extracted from heart tissue, analyzed by iTRAQ (Additional file 11: Table S9; data includes proteins with at least a 1.3-fold change in expression when compared to time-matched controls), and compared with gene expression at all three time points. Proteins and gene expression data were largely discordant at T_c,Max_ (Figure 4). This time point had significant enrichment in genes encoding proteins involved in the heat-stress response, especially the HSPs (Figure 4, genes in bold). However, there were too few differentially expressed proteins at T_c,Max_ to derive functional clusters (Additional file 11: Table S9). Although HSP70 and the gene encoding this protein were the most upregulated gene/protein pair at T_c,Max_, HSPB1 was the only other heat shock protein modulated in the protein data at this time point, and its expression decreased while the transcript encoding the protein product increased. Proteins associated with serine proteinase activity were upregulated, including α-1 macroglobulin precursor and the acute phase protein α-1-inhibitor 3 precursor (MUG2, A1I3), but the transcripts for these products were below the fold-change threshold. Both proteins are serum proteins and changes may reflect changes blood contamination not necessarily changes in the heart tissue itself. Further, several mitochondrial proteins were modulated, including mitochondrial precursor hydroxyacyl-coenzyme A dehydrogenase and mitochondrial import inner membrane translocase subunit, but the transcript expression did not correlate with the protein expression.

**Figure 5:**
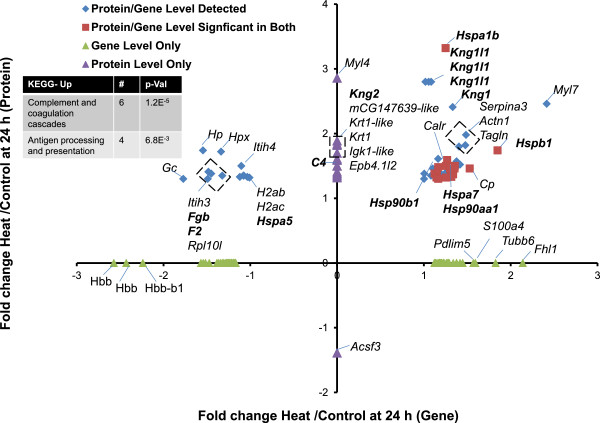
**Differentially expressed genes and proteins and KEGG pathway enrichment analysis at 24 hours.** Proteins were mapped to gene probes to compare differentially expressed proteins and genes in the heart. Insets show the results of the KEGG pathway enrichment analysis for concordant gene and protein up- and down-regulation. Genes and proteins with the greatest concordance with the pathway enrichment analysis are depicted. Proteins expressed at ±1.3-fold and their respective genes (±2-fold) are represented by red squares. Proteins and genes which mapped together but did not meet significance at the gene level are represented as blue diamonds. Genes (green triangles) or proteins (purple triangles) that were expressed and significant, but did not map together, are plotted along the axes. For clarity, only the gene designations are listed.

At 24 hours, 88 proteins were significantly altered (Additional file 11: Table S9), but few protein/gene expression pairs were in concordance (HSPA1B and its corresponding transcript; Figure 5). By 24 hours, multiple HSPs were expressed above the fold-change threshold (i.e., HSPA5, HSP90B1, HSPB1, HSPA7, and HSP90AA1; bold in Figure 5). However, all of the transcripts encoding the HSPs which had been up-regulated at T_c,Max_ were either down-regulated (HSPA5*)* or below the fold-change threshold, with the exception of HSPB1 (Figure 5). HSP90AA1, HSP90B1, and calreticulin precursor (CALR) clustered to the functional KEGG pathway Antigen Processing and Presentation (Figure 5, inset), although their respective transcripts did not reach the fold-change threshold (Figure 5). The enrichment of the Complement and Coagulation cascades KEGG pathway was driven by up-regulation of the proteins KNG1, KNG2, KNG1L1, C4, F2, and FGB, although there was not a complete correlation between transcript/protein expression level and direction (Figure 5).

Thus, at 24 hours environmentally inducible HSPs remained elevated, despite a lack of blood chemistry or histopathologic evidence of injury. The HSPs elevated at 24 hours are associated with cardiomyocyte injury and immune-mediated functions (e.g., Antigen Processing and Presentation). HSPA5 is an ER-resident chaperone associated with hypoxia in cardiomyocytes [81, 82]. HSPA5 retargets to mitochondria and contributes to the mitochondrial unfolded protein response [83]. Extracellular HSPs associated with Antigen Processing and Presentation have been associated with immune cell recruitment to cardiomyocytes, a potential mechanism of inflammatory injury [50].

**Figure 6:**
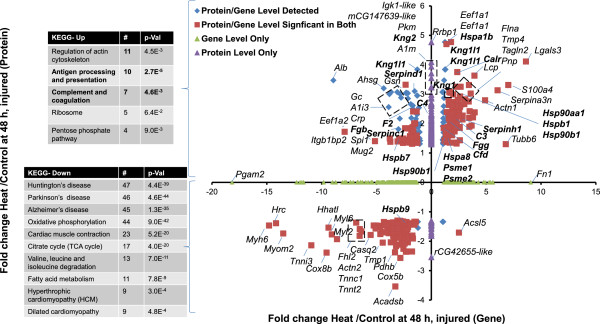
**Differentially expressed genes and proteins and the KEGG pathway enrichment analysis in hearts** ***with*** **evidence of histopathological injury 48 hours after heat stress.** Proteins were mapped to gene probes to compare differentially expressed proteins and genes in the heart. The KEGG pathway analysis results for genes and proteins with the greatest concordance are depicted. Heat shock proteins and proteins related to HSPs are bolded. Proteins expressed at ±1.3-fold and their respective genes (±2-fold) are represented by red squares. Proteins and genes which mapped together but did not meet significance at the gene level are represented as blue diamonds. Genes (green triangles) or proteins (purple triangles) that were expressed and significant, but did not map together, are plotted along the axes. For clarity, only the gene designations are listed.

**Figure 7:**
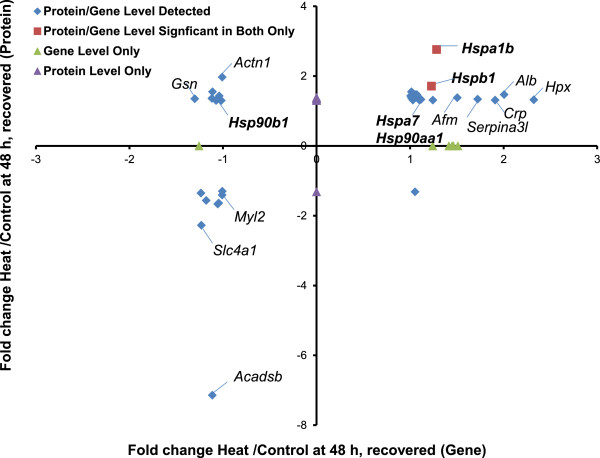
**Differentially expressed genes and proteins in uninjured hearts 48 hours after heat stress.** Proteins were mapped to gene probes to compare differentially expressed proteins and genes in animals with uninjured hearts. Proteins expressed at ±1.3-fold and their respective genes (±2-fold) are represented by red squares. Proteins and genes which mapped together but did not meet significance at the gene level are represented as blue diamonds. Genes (green triangles) or proteins (purple triangles) that were expressed and significant, but did not map together, are plotted along the axes. For clarity, only the gene designations are listed.

**Figure 8:**
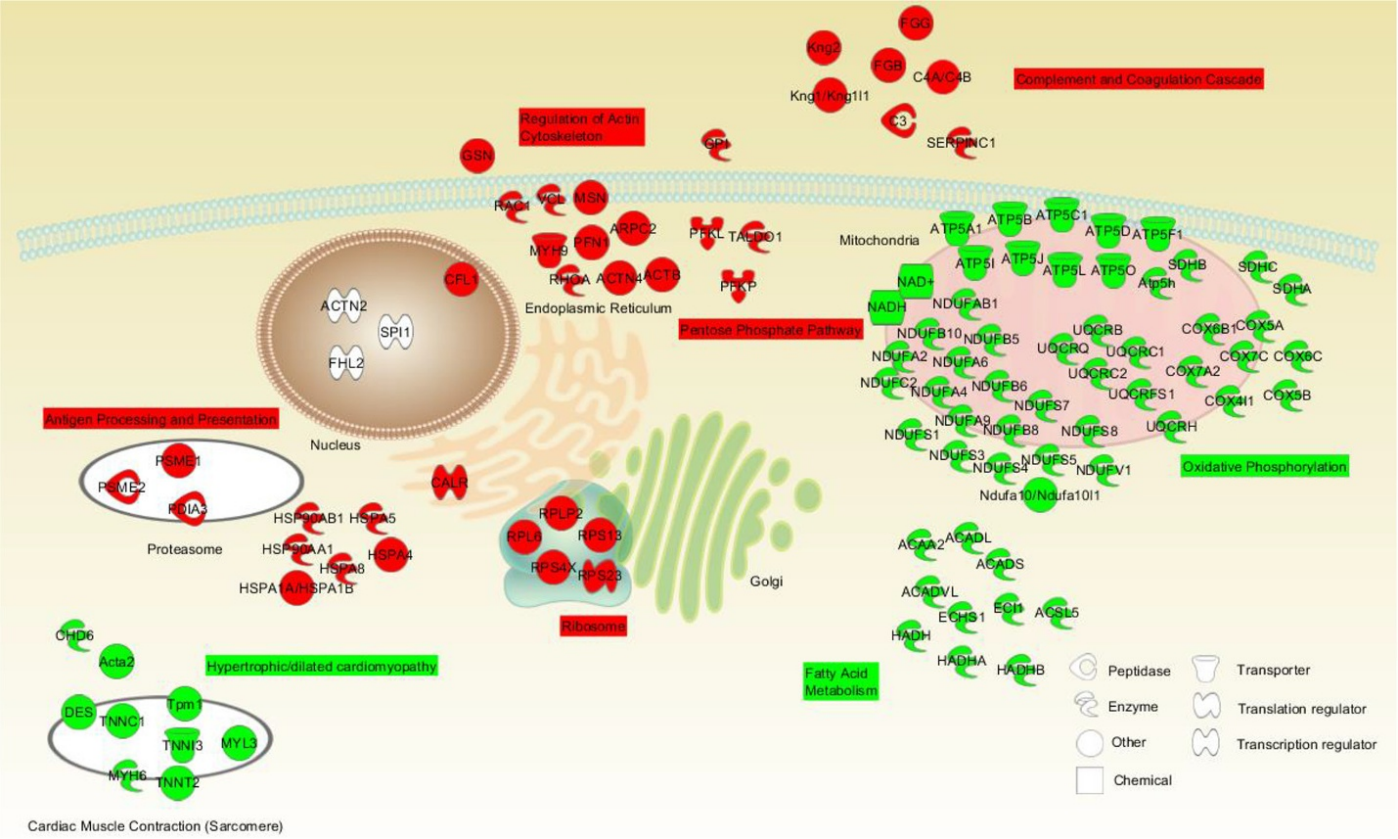
**Aggregation propensities for modulated pathways.** Proteins and genes with concordant differential expression in heat-injured animals at 48 hours were mapped to subcellular localization (see Figure 6). Up-regulated proteins/gene pairs mapped to the KEGG pathways Ribosome, Antigen Processing and Presentation, Regulation of Actin Cytoskeleton, Complement and Coagulation Cascade, and Pentose Phosphate Pathway. Down-regulated protein/gene pairs mapped to Hypertrophic/Dilated Cardiomyopathy and Cardiac Muscle Contraction, Fatty Acid Metabolism, and Oxidative Phosphorylation. Gene/protein pairs are mapped to target subcellular organelles. For simplicity in presentation, only protein designations are shown.

At 48 hours, we subdivided the animals based on histopathologic evidence of injury (Figure 6) or recovery (Figure 7). Five hundred and twenty-one proteins were significantly altered in animals with histopathological evidence of heart injury (Figure 6 and Additional file 11: Table S9), whereas only 71 were modulated in heat-stressed animals with no histopathological evidence of injury (Figure 7 and Additional file 11: Table S9). The up-regulated proteins were enriched for pathways associated with protein folding (Ribosome; Figure 6, inset) and, by extension, propensity to aggregate [80]. Pathways associated with immune dysregulation were enriched (Complement and Coagulation, including concordant transcript and protein expression of KNG1, KNG1L1, KNG2, C3, C4, CFD, F2, FGB, FGG, SERPINC1, SERPIND1, and SERPINH1; and Antigen Processing and Presentation, including concordant protein expression of HSPA1B, HSP90AA1, HSPA5, CALR, HSPA8, and HSPAB1). HSP expression correlated with transcript expression only in the heat-injured animals (red squares in Figure 6), not the heat-stressed animals (Figure 7). Down-regulation of pathways enriched for Oxidative Phosphorylation, citrate cycle, and fatty acid metabolism suggested an energy crisis with massive mitochondrial dysfunction, supporting the observed necrosis in the cardiac histopathology. In addition, KEGG enrichment analysis of down-regulated proteins identified pathways associated with chronic proteotoxic injury (Parkinson’s Disease, Huntington’s Disease, Alzheimer’s Disease; Figure 6, inset) [79]. Protein/gene pairs which showed concordant regulation in 48 hour heat-injured animals were diagrammed by subcellular organelle (Figure 8 and Additional file 12: Table S10 and Additional file 13: Table S11). Most of the protein/gene pairs associated with mitochondria were associated with the Oxidative Phosphorylation, with nearly 100% overlap with protein/gene pairs in the KEGG pathways of Parkinson’s Disease, Huntington’s Disease, and Alzheimer’s Disease. Likewise, the heat shock proteins and proteins/gene pairs associated with proteasomal degradation were localized to the proteasome, including biomolecules in the immunomodulatory pathway Antigen Processing and Presentation. The Cardiomyocyte Contraction pathway contained protein/gene pairs which were downregulated in conjunction with the sarcomere.

### Protein aggregation and supersaturation by subcellular localization in heart tissue

**Figure 9:**
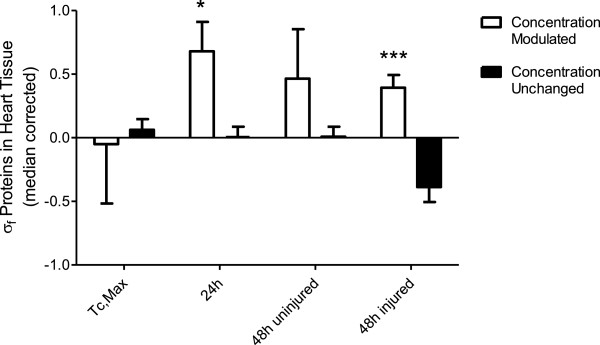
**Preferential increase in propensity to aggregate in proteins modulated by heat stress at 24 and 48 hours with histopathological evidence of cardiac injury.** Median-corrected supersaturation scores (σ_f_) were calculated for proteins which changed (white bars) or remained unchanged in abundance as measured by mass spectrometry after heating as described by Ciryam *et al.*[79].

Supersaturation (i.e., high cellular concentration of proteins with a high propensity to aggregate) of proteins has been associated with some of the KEGG pathways we found modulated by heat stress. Many of the KEGG pathways we identified as enriched in heat-injured animals at 48 hours have been associated with neurodegenerative protein misfolding disorders, including Oxidative Phosphorylation (and the related pathways Alzheimer’s Disease, Parkinson’s Disease, and Huntington’s Disease), Cardiomyocyte Contractility, and Proteasome [79]. In addition, proteins in these biochemical pathways have a combined high propensity to aggregate and elevated concentrations in proteotoxic diseases characterized with protein misfolding [79]. Because heat induces protein misfolding and dysregulation, we investigated whether proteins affected by heat stress and heat injury had higher propensities to aggregate as a function of subcellular organelle localization (Figure 9 and Additional file 12: Table S10).

The supersaturation scores for proteins modulated after heating at 24 hours and at 48 hours in heat-injured animals (Figure 9; *p*<0.05), but not at T_c,Max_, was higher than for proteins whose abundance was unchanged at these time points. Average supersaturation score of proteins modulate in animals without evidence of cardiac injury at 48 hours was also higher, but was not significantly different from the average for the unchanged proteins (in part due to a smaller number of proteins changing in abundance at 24 hours vs 48 hours). These data indicate that: (1) preferential modulations in abundance of proteins with high supersaturation scores occur as early as 24 hours; and (2) proteins prone to aggregation are more likely to be regulated in cardiac injured rats than in animals that do not show histopathological evidence of injury at 48 hours. This suggests that protein aggregation is contributing to changes in protein abundance and perturbation of the biological processes associated with these proteins. Further, aggregation as a result of heating is possibly a leading mechanism in the heat injury observed at 48 hours.

## Conclusion

Here, we present a global characterization of gene and protein response to heat stress in a conscious rat model that provides new insight into the regulatory processes associated with heat stress and recovery. *1*) Transcriptional analyses of the heart, lung, liver, and kidney at T_c,Max_ revealed a consensus heat stress-induced gene response that was enriched for genes related to protein folding and the regulation of apoptosis. *2*) We used self-organizing maps to group genes into clusters of similar expression profiles in animals with evidence of similar histopathologies. In the hearts of three animals at 48 hours, significantly enriched Oxidative Phosphorylation, Parkinson’s Disease, Huntington’s Disease, Antigen Processing and Presentation, and Cardiac Muscle Contractility KEGG pathways were consistent with protein-folding disorders, mitochondrial dysfunction, and perturbation in cellular energetics. *3*) Transcriptional responses were corroborated by the results of proteomic analysis in heat-injured cardiac tissue at 48 hours, with pathway analysis, protein aggregation scores, and subcellular localization supporting up-regulation of proteins and genes in Ribosome, Antigen Processing and Presentation, Regulation of Actin Cytoskeleton, Complement and Coagulation Cascade, and Pentose Phosphate Pathway, and downregulation of Hypertrophic/Dilated Cardiomyopathy, Fatty Acid Metabolism, and Oxidative Phosphorylation (Figure 8). Proteins involved in Antigen Processing and Presentation emerge as early as 24 hours. Disruption in these mitochondrial mediators and deficits in pathways related to energetics precede large changes in mediators of oxidative phosphorylation and protein folding disorders by 48 hours in heat-injured cardiac tissue. *5*) Supersaturation scores indicated an increased propensity to aggregate in proteins changing in abundance after heat stress at 24 hours and 48 hours. Increased propensity to aggregate at 48 hours in animals with histopathological evidence of heat injury was more pronounced than in animals without cardiac injury. Disruption in protein folding and the resulting aggregation is likely to contribute to the persistent subcellular indicators of energy crisis and regulation of apoptosis observed in heat-injured animals.

The Fischer 344 strain was chosen because they do not gain body weight as quickly as Sprague–Dawley, which can have a large impact on thermal dynamics. It is possible that naturally occurring lesions in the heart were exacerbated by heat stress, however, only those with severe lesions were correlated with animals that demonstrated both gene and protein alterations (i.e. uninjured animals, with or without background lesions, were indistinguishable at the molecular level). It is important to note that many humans have pre-existing conditions leading to heat susceptibility, and it is possible to extrapolate our findings to these populations. Given that most heat stroke deaths occur in the elderly, this would be particularly relevant to those populations, although not exclusive. Future studies should attempt to corroborate the findings of our study using rat strains less susceptible to the congenital cardiomyopathy phenotype. Further, intermediate time points between T_c,Max_, 24 hours, and 48 hours should be investigated to more accurately chronicle the initiation and progression of organ injury in the heart. However, our model may prove useful in identifying and understanding mediators of heart injury after heat stress in susceptible individuals.

This work in heat-stressed animals provides the basis for an assessment of global gene and protein responses in future heat-stroke–induced organ injury experiments. Top-down integration of heat-induced changes in metabolomics, proteomics, and transcriptomics (to include small RNA) will provide the foundation for a computationally based linkage to human three-dimensional thermoregulation models. Anchoring these systemic stress responses to the physiological model of heat stroke will provide further insight into predicting risk, severity, and timing of organ injury in response to hyperthermia. Such a model will potentially accelerate the development of strategies to improve prevention, classification, and treatment of heat-related illnesses.

## Methods

### Animal model and tissue collection

*In vivo* rat experiments were performed at the US Army Research Institute of Environmental Medicine. The Institutional Animal Care and Use Committee approved all experimental procedures, which complied with the American Physiological Society’s guiding principles for research involving animals and the Guide for Care and Use of Laboratory Animals. As previously described [20], we utilized male Fischer 344 rats (F344; n = 36; Charles River Laboratories, Stone Ridge, NY) weighing 234–336 g (~2–3 months old). Briefly, rats were housed under standard laboratory conditions (22°C, 12:12 hours light:dark cycle, lights on at 6:00 AM) in an Association for Assessment and Accreditation of Laboratory Animal Care–accredited facility. We provided chow (Harlan Teklad, LM-485, Madison, WI) and water *ad libitum* [84]. Rats were implanted with TL11M2-C50-PXT PhysioTel® Multiplus Transmitters (Data Sciences International, St. Paul, MN) to measure core temperature (T_c_; ±0.25°C), heart rate (HR; bpm, beats per minute), and mean arterial pressure (MAP; ±3 mm Hg).

We conducted all experiments in conscious, free-moving animals, as previously described [20]. We used 36 rats with remote telemetry implants for this study (Figure 1). Briefly, we placed rats in the heat-stress arm of the experiment into a floor-standing incubator (Thermo Scientific, Ashville, NC) set at room temperature (22°C) 24 hours prior to initiation of heat stress experiments. Rats in the control arm (n=18) were never introduced to the incubator environment and were kept at a normal housing temperature. Once T_c_ ≤ 37.3°C was measured (Figure 1), we weighed the control rats and returned them to their original cages for the remainder of the experiment. Rats in the heat-stress arm were heated in the incubator at 37.0 ± 0.2°C until reaching a T_c_ of 41.8°C (T_c,Max_). Six heat-stressed rats were weighed at T_c,Max_ and euthanized as previously described [20]. The remaining rats were placed in a new cage at the ambient housing temperature (22.0 ± 0.2°C) and euthanized at 24 hours (n=6) or 48 hours (n=6) (Figure 1). Six time-matched controls were euthanized at times corresponding to T_c,Max_, 24 hours, and 48 hours in the heat-stress arm. Time points were chosen to maximize the likelihood of observing changes in gene expression and to reflect the clinical observation that recovery from heat exhaustion usually takes 24 to 48 hours following mild injury. We provided control and experimental animals with food and water *ad libitum* throughout recovery.

### Physiological and hematological parameters

We measured body weight (BW) on a top-loading balance with an accuracy of ±0.1 g. The difference between pre- and post-heat BW was used as an indicator of percent dehydration using the following calculation: [(post–heat-stress BW – pre–heat-stress BW)/pre–heat-stress BW] × 100. We did not take into account changes in body weight due to urine or feces loss. Rats were deeply anesthetized under isoflurane anesthesia, and a thoracotomy was performed, followed by a cardiac puncture to collect blood for hematologic analysis. We determined hemoglobin (hgb) and hematocrit (hct) using a VetScan HM5 Hematology System (Abaxis, Union City, CA), and BUN (mg/dL) using a handheld iStat clinical analyzer (Abbott Laboratories, Abbott Park, IL) and 95 μL of the collected blood. We placed the remaining blood in heparinized tubes and immediately stored it on ice. Plasma was isolated via centrifugation (4°C; 5 minutes, ~2,000 rpm), placed in aliquots, and stored at -80°C until assays were performed. We determined plasma alanine aminotransferase (ALT) (U/L) and aspartate aminotransferase (AST) levels (U/L) in duplicate using the Poly-Chem® System (Polymedco Inc., Cortlandt Manor, NY) per manufacturer specifications. Precision of the ALT and AST assays were valid to a minimum of ±10 U/L and ±12.1 U/L, respectively. Statistical significance among groups was determined by Kruskal-Wallis analysis of variance with post-hoc Tukey’s honest significant differences test relative to control, with an α of 0.05 denoting statistical significance.

### Histopathology

Fixed tissues (heart, liver, lung, and kidney) were mounted and hematoxylin and eosin (H&E)-stained (IHC World, Woodstock, MD). A total of 24 rats were separated into three groups: T_c,Max_, 24 hours recovery, and 48 hours recovery. Each group comprised six heat-stressed and two time-matched control animals. We performed serial sections to produce 20 unstained slides per tissue, with three to five sections per slide. Experimental Pathology Laboratories® (Sterling, VA) conducted the histopathological evaluation. The magnitude of inflammatory or degenerative lesions was graded on a scale of 1 to 5, with grade 1 being minimal and grade 5 being severe (See Additional file 1: Table S1, Additional file 2: Table S2, Additional file 3: Table S3).

### Isolation of RNA

Frozen tissues (heart, lung, liver, and kidney) stored at -80°C in cryovials were placed on dry ice, cut into aliquots with a sterile scalpel on a pre-chilled titanium block, and placed in new tubes pre-chilled on dry ice. The block was washed with RNase *Zap* (Ambion, Life Technologies, Grand Island, NY) between samples. While working on dry ice, whole tissues (~25 mg) from the lung, liver, and kidney were cut from each sample and placed in 700 μl of QIAzol Lysis Reagent. Samples were homogenized with a TissueLyzer LT (Qiagen, Valencia, CA) for 5 minutes at 25 Hz twice, allowed to sit at room temperature for 5 minutes, then placed at -80°C.

We homogenized heart tissue with a 6750 Freezer/Mill with three sample microvials (Spex SamplePrep, Metuchen, NJ). Approximately 1 cm^3^ of heart tissue was cut, dipped into liquid nitrogen, and then placed into pre-assembled and pre-chilled microvials containing 200 μL of QIAzol. An impactor was added to each microvial, securely capped, and placed in the mill. The Freezer/Mill settings were as follows: T_1_ run time = 3 min, T_2_ intermission = 1 min, T_3_ pre-cool = 5 min, rate = 15, and cycles = 2. After completion, we added 500 μL of QIAzol to each vial, allowed the vial to sit at room temperature for 5 minutes, transferred the sample to a microfuge tube, and then placed it at -80°C.

The following day, we allowed all samples to thaw and isolated the RNA using the miRNeasy 96 kit (Qiagen), according to manufacturer’s instructions. The quality and quantity of RNA samples were evaluated with a 2100 Bioanalyzer (Agilent Technologies, Santa Clara, CA), using the Agilent RNA 6000 Nano Reagents, and a multiwell Nanodrop 8000 spectrophotometer (Thermo Fisher Scientific, Waltham, MA).

### Affymetrix gene array

We prepared cDNA from total tissue RNA using the Ambion Whole Transcript (WT) Expression Kit (Ambion) according to manufacturer’s recommendations, fragmenting the cDNA using the GeneChip WT Terminal Labeling Kit (Affymetrix, Santa Clara, CA). Following manufacturer’s instructions, we prepared labeled cDNA, trays, and arrays with the GeneTitan Hybridization, Wash and Stain Kit for WT array plates (Affymetrix). Samples were then applied to the Rat Gene 1.1 ST 16 Array Plate or 24 Array Plate and placed in the GeneTitan System (Affymetrix), according manufacturer’s recommendations. Of the 144 arrays, 2 liver, 1 lung, and 1 heart array did not pass the GeneTitan scanning quality control. Thus, 4 arrays (2.7%) were excluded from further analysis.

### Gene array data analysis

For each organ, we normalized arrays using an extension of the PLIER (Probe Logarithmic Intensity Error) algorithm, called the iterPLIER procedure, in the Affymetrix Expression Console [84]. The iterPLIER (exon level) procedure discards feature sets which perform poorly, as described by Qu *et al.* [85]. We imported the resulting CHP files into Partek. Affymetrix library files included all available reference files related to RaGene-1_1-st-v1.na32.rn4. To address signal variance following the iterPLIER analysis, we applied a variance-stabilizing transformation (PLIER+16) to raw intensity (Guide to PLIER Estimation) [84]. Standard deviation for each transcript ID were determined across all samples for each organ, and we removed those lower than the median and retained the remaining transcript IDs for determination of differentially expressed genes and as background for our pathway analysis of each organ. The low-variance criterion from Bourgon *et al.* [86] was implemented by computing and sorting the expression variance of each gene over the complete condition set, then removing the low-variance genes (lower half). To determine DEGs, a two-way analysis of variance (ANOVA) was conducted for each organ, and gene lists were generated by applying a two-fold change cutoff with the Benjamini-Hochberg False Discovery Rate (FDR <0.05) [87, 88]. Microarray data described in this study have been deposited in the Gene Expression Omnibus database with accession number GSE56740.

### Gene Set enrichment analysis

DEGs were analyzed for enriched GO terms using Partek. We considered only terms with <500 members and removed terms with <4 members identified. Additionally, only terms with enrichment scores above two-fold with *p*-values below 0.05 were reported. Gene lists were examined with the gene set analysis tools in Partek software and DAVID [24, 25]. Enriched KEGG pathways and FACs (functional annotation clusters) were determined using default settings for each organ. Transcript IDs from functionally associated clusters with an enrichment score >2.0 were consolidated and redundant genes removed. Gene lists were consolidated and sub-categorized to account for redundant annotation terms comprising FACs. For representation purposes, unique transcripts were consolidated from similar FACs.

### ITRAQ proteomic analysis

We dried 100 μg of protein in a centrifugal evaporator and prepared the sample for mass spectrometry according to the iTRAQ reagent protocol (SCIEX, Framingham, MA). Samples were reduced in 50 mM tris-(2-carboxyethyl) phosphine, cysteines were blocked in 200 mM methanethiosulfonate, and proteins were digested in 5 μg trypsin overnight. We fractionated the samples using a Polysulfomethyl A column (200 × 4.6 mm, 5 μm, 1000 Å, PolyLC, Columbia, MD) at a flow rate of 0.95 μL/min. An Agilent 1100 high performance liquid chromatography (HPLC) equipped with an autosampler with an expanded injection loop and needle seat, diode array detector, fraction collector and Chemstation data system was used for the separation. The column compartment was set to 35°C, and 1 mL of the sample was injected. The solvents used for the separation were solvent A (distilled water), solvent B (500 mM ammonium formate, and 3% formic acid in water), and solvent C (acetonitrile). The initial conditions were 73% A: 2% B: 25% C. A linear gradient was performed to 75% B: 25% C in 20 minutes, with a stop time of 30 min. One-minute fractions were collected starting at 4 minutes and continuing until 19 minutes, producing 15 fractions. Samples were dried and reconstituted in 80 μL of 0.1% formic acid. Following reconstitution, fractions 1, 13, 14, and 15 were combined into one sample, dried, and reconstituted again in 80 μL of 0.1% formic acid.

We separated the peptides using a Thermo Scientific Proxeon EASY nano-LC prior to direct injection into a Thermo Orbitrap Velos mass spectrometer (ThermoScientific Corp, West Palm Beach, FL). The peptides were trapped using an Agilent Zorbax C-18 5 × 0.3 mm, 5 μm particle size column. The analytical separation was performed using a Waters NanoAcquity UPLC column BEH C18 100 μm × 100 mm, 1.7 μm particle. Ten microliters of the sample were loaded on the column and flushed with 60 μL of 0.1% formic acid in water. The solvents used were 0.1% formic acid in water (Buffer A) and 0.1% formic acid in acetonitrile (Buffer B) were used. The gradient profile was as follows: initially 3% B, 45% B at 90 minutes, 95% B at 100 minutes, 95% B at 115 minutes, 3% B at 116 min, and the analysis stopped at 120 minutes. The flow rate was 0.30 μL/min. Precursor selection was done in a Thermo Orbitrap using 100,000 resolution. The top 10 peptides, based on intensity, were selected for fragmentation. Sample sets were analyzed with a single scan, using stepped higher-energy collisional dissociation from 40 and 50 V. Species with single or unidentified charge states were excluded from precursor ion selection. Dynamic exclusion was used with a 20-second window.

We processed mass spectral data using Thermo Proteome Discoverer 1.3 (ThermoFisher Scientific) and searched for samples using the Sequest-generated randomized rat database based on all *Rattus norvegicus* sequences found in the National Center for Biotechnology Information (NCBI) Reference Sequence Database (4-12-2012), with the mass tolerance values set at 10 ppm for precursor ions and 0.8 Da for fragment ions [89]. Other parameters included fixed-modification methylthio (cysteine) and iTRAQ™ 8-plex (N-terminus/lysine). The variable modifications allowed were oxidation (methionine), phosphylation (serine/tyrosine/threonine), acetylation (histidine/serine/tyrosine/threonine), modification by N-acetylglucosamine (asparginine/serine/threonine), modification by hexose-linked N-acetylglucosamine (serine/threonine) and iTRAQ™ 8-plex (tyrosine). Scans having peptide identifications with a 1% FDR were used for quantification. Reporter ions were quantified with a mass tolerance window of 20 ppm using the most confident centroid. Reporter ion intensities were corrected using the Discoverer 1.3 default method of iTRAQ™ 8-plex mass tags by Applied Biosystems optimized for ThermoFisher Scientific.

### Quantitative iTRAQ data analysis

To quantitate peptide and protein identifications from MS/MS spectra, we used Scaffold Q+ (version Scaffold 4.2.1, Proteome Software Inc., Portland, OR). Peptide identifications of greater than 95.0% probability by the Scaffold Local FDR algorithm were accepted, and protein identifications of greater than 99.0% probability with at least two identified peptides and probabilities were assigned by the Protein Prophet algorithm [90]. To satisfy the principles of parsimony, we grouped into clusters the proteins containing similar peptides not differentiable by MS/MS analysis alone, correcting channels as described in i-Tracker [91]. Acquired intensities in the experiment were globally normalized across all acquisition runs, and individual quantitative samples were normalized within each acquisition run. We normalized intensities for each peptide identification within the assigned protein, and normalized the reference channels to produce a 1:1 fold change. All normalization calculations were performed using medians to multiplicatively normalize data. Differentially expressed proteins were determined using a ±1.3-fold threshold as described by Ahn *et al.* [92].

### Calculation of supersaturation scores

Supersaturation scores were calculated based on the methods of Ciryam *et al.* [79]. Briefly, supersaturation scores are the sum of propensity to aggregate and the log of the protein concentration normalized to the median score for all proteins detected. The propensity to aggregate for unfolded proteins (Z_agg_) and proteins in their native state (Z^SC^_agg_) were kindly calculated by Tartaglia and Venduscolo for all proteins identified by our MS analysis [92]. Protein abundance was estimated based on the sum of the total ion intensity for the top three most abundant peptide ions, which has been shown to correlate well with protein abundance [93]. Precursor ion intensities determined by extracted ion chromatograms in Proteome Discoverer were summed across all fractions and charge states. For peptides where multiple precursor intensities were reported within a fraction, only the highest intensity was used. Only proteins with three identified peptides were used in this analysis. Total ion intensity for all samples in a single iTRAQ experiment was converted to sample ion intensities using the protein ratio data from Scaffold. Ion intensity were converted to protein concentrations using the known concentration of human homologs MB, HBB, CKM, MYL2, GAPDH, FABP3, and LDHB [94]. Average supersaturation scores were calculated for proteins whose abundance changed (>±1.3 fold) or didn’t change (<±1.1) between an experimental condition and the time matched control. A one way ANOVA with post hoc Bonferroni correction Statistics was conducted in Graphpad Prism (v5). Data is plotted as mean +/- standard error of the mean.

## Electronic supplementary material

Additional file 1: Table S1: Table summarizing the blood chemistry data. (DOCX 17 KB)

Additional file 2: Table S2: Table listing the histopathologies in animals at T_c,Max_. (DOCX 19 KB)

Additional file 3: Table S3: Table listing the histopathologies in animals 24 hours after heat stress. (DOCX 18 KB)

Additional file 4: Figure S1: Figure illustrating the histopathological evidence of kidney and liver injury that occurred in one rat 24 hours after heat stress. (DOCX 1 MB)

Additional file 5: Table S4: Table of histopathologies 48 hours after heat stress. (DOCX 19 KB)

Additional file 6: Table S5: Table of all differentially expressed genes in the heart, liver, lung, and kidney at T_c,Max_, 24 hours, and 48 hours. (XLSX 214 KB)

Additional file 7: Table S6: Table listing the enriched GO terms by organ at T_c,Max_, 24 hours, and 48 hours. (XLSX 209 KB)

Additional file 8: Table S7: Table listing DEGs mapping to KEGG pathways and genes of interest identified by self-organizing maps for both the heart (48 hours) and liver (24 hours). (XLSX 97 KB)

Additional file 9: Table S8: Table listing the functional annotation clusters identified by DAVID analysis. (XLSX 43 KB)

Additional file 10: Figure S2: Figure showing the self-organizing maps identifying gene nodes enriched in the liver at 24 hours. (DOCX 458 KB)

Additional file 11: Table S9: Table of differentially expressed proteins (DEP) identified by quantification of normalized intensities by iTRAQ proteomics. (XLSX 2 MB)

Additional file 12: Table S10: Table listing supersaturation score calculations. (XLSX 2 MB)

Additional file 13: Table S11: Table listing the KEGG pathways modulated in proteins after heat stress. (XLSX 18 KB)

## Abbreviations

AIRAP: Arsenite-inducible RNA-associated protein
ALT: Alanine aminotransferase
ANOVA: Analysis of variance
AST: Aspartate aminotransferase
*Atf3*: Activating transcription factor 3
BP: Biological processes
BUN: Blood urea nitrogen
BW: Body weight
CALR: Calreticulin precursor
CC: Cellular component
cHSR: Consensus heat stress response
CREB: Cyclic AMP response element-binding
DAVID: Database of Annotation, Visualization and Integrated Discovery
DEG: Differentially expressed genes
ER: Endoplasmic reticulum
FAC: Functional annotation clusters
FDR: False discovery rate
GO: Gene ontology
Hadh: Hydroxyacyl-coenzyme A dehydrogenase
hct: Hematocrit
HD: Huntington’s disease
hgb: Hemoglobin
HR: Heart rate
*Hsf1*: Heat shock factor 1
HSP: Heat shock protein
ID: Identifier
iTRAQ: Isobaric tag for relative and absolute quantitation
KEGG: Kyoto Encyclopedia of Genes and Genomes
LPS: Lipopolysaccharide
MAPK: Mitogen-associated protein kinase
MF: Molecular functions
NCBI: National Center for Biotechnology Information
OP: Oxidative phosphorylation
P: Proteasome
PD: Parkinson’s disease
PF: Protein folding
PLIER: Probe logarithmic intensity error
RA: Regulation of apoptosis
SIRS: Systemic inflammatory response syndrome
TLR: Toll-like receptor.
