# Supplementary material for: Patterns of gene expression associated with recovery and injury in heat-stressed rats
Source: BMC Genomics. 2014 Dec 3;15(1):1058. doi: 10.1186/1471-2164-15-1058 (PMC4302131; doi:10.1186/1471-2164-15-1058)
Supplement: Supplementary file 1 — Additional file 1: Table S1: Table summarizing the blood chemistry data. (DOCX 17 KB) [file 12864_2014_6768_MOESM1_ESM.docx]

|  | **Control** | **Heat, T_c,Max_** | **Heat, 24 h** | **Heat, 48 h** |
| --- | --- | --- | --- | --- |
| **Parameter** | **Mean ± SD** | **Mean ± SD** | **Mean ± SD** | **Mean ± SD** |
| Change in body weight (% control) | 100.0 ± 0% | 92.4 ± 1.6% * | 92.1 ± 12.7% | 99.0 ± 3.0% |
| ***Hematology*** | | | | |
| White blood cells (10^9^/L) | 5.1 ± 2.0 | **2.0 ± 0.9 *** | 6.5 ± 1.5 | 5.2 ± 0.9 |
| Red blood cells (10^12^/L) | 7.5 ± 1.1 | **9.5 ± 0.5 *** | 8.1 ± 0.5 | 8.1 ± 0.5 |
| Hemoglobin (g/dL) | 12.8 ± 2.4 | **16.3 ± 0.9 *** | 14.0 ± 0.4 | 14.0 ± 0.9 |
| Hematocrit (%) | 37.4 ± 5.5 | **46.9 ± 3.1 *** | 41.5 ± 1.6 | 41.0 ± 3.1 |
| ***Liver/Kidney Function*** | | | | |
| Alkaline phosphatase (ALP) (U/L) | 274.9 ± 28.0 | 280.3 ± 25.7 | 237.0 ± 36.4 | 241.2 ± 25.7 |
| Alanine aminotransferase (ALT) (U/L) | 66.8 ± 10.2 | 97.0 ± 20.2 | 124.7 ± 98.7 | 93.4 ± 20.2 |
| Total bilirubin (mg/dL) | 0.2 ± 0.0 | 0.2 ± 0.1 | 0.3 ± 0.1 | 0.3 ± 0.1 |
| Albumin (g/dL) | 4.6 ± 0.6 | **5.9 ± 0.5 *** | 3.6 ± 0.4 | 4.4 ± 0.5 |
| Blood urea nitrogen (mg/dL) | 15.2 ± 1.7 | **28.5 ± 3.3 *** | 14.7 ± 2.5 | 14.6 ± 3.3 |
| ***Electrolytes*** | | | | |
| Na (mmol/L) | 137.4 ± 0.8 | **146.8 ± 4.1 *** | 137.3 ± 1.0 | 137.7 ± 4.1 |
| K (mmol/L) | 3.9 ± 0.3 | 4.5 ± 0.8 | 4.2 ± 0.3 | 4.4 ± 0.8 |
| Cl (mmol/L) | 99.4 ± 0.9 | **107.0 ± 6.2 *** | 101.0 ± 2.2 | 99.7 ± 6.2 |
| *, p<0.05 by Kruskal-Wallis analysis of variance with post-hoc Tukey HSD [honest significant differences] test relative to control (bold); SD, standard deviation | | | | |

**Additional File 1.** Summary table of blood chemistries in control and heat-stressed rats
