# Supplementary material for: Patterns of gene expression associated with recovery and injury in heat-stressed rats
Source: BMC Genomics. 2014 Dec 3;15(1):1058. doi: 10.1186/1471-2164-15-1058 (PMC4302131; doi:10.1186/1471-2164-15-1058)
Supplement: Supplementary file 2 — Additional file 2: Table S2: Table listing the histopathologies in animals at Tc,Max. (DOCX 19 KB) [file 12864_2014_6768_MOESM2_ESM.docx]

**Additional File 2.** Histopathological findings in rats at T_c,Max_

|  | Animal Number | | | | | | | |
| --- | --- | --- | --- | --- | --- | --- | --- | --- |
|  | Heat-Stressed | | | | | | Control | |
|  | T_c,Max_ 7 | T_c,Max_ 8 | T_c,Max_ 9 | T_c,Max_ 10 | T_c,Max_ 11 | T_c,Max_ 12 | T_c,Max_ 1 | T_c,Max_ 2 |
| **Heart** |  |  |  |  |  |  |  |  |
| Cardiomyopathy | 1 | 0 | - | - | - | 1 | 1 | - |
| Inflammation, subacute, with cardiomyocyte degeneration and loss | - | 0 | - | - | - | - | - | - |
| **Kidney** |  |  |  |  |  |  |  |  |
| Chronic progressive nephropathy | - | 1 | - | - | 1 | - | 3 | - |
| Pelvic dilatation | - | - | - | - | - | - | - | 4 |
| Necrosis, tubular, diffuse, acute | - | - | - | - | - | - | - | - |
| Proteinosis | - | - | - | - | - | - | - | - |
| Proteinosis, tubular, diffuse | - | - | - | - | - | - | - | - |
| **Liver** |  |  |  |  |  |  |  |  |
| Inflammation, chronic | 1 | 1 | - | 1 | - | - | 1 | - |
| Pigment, intracellular | - | 2 | 2 | - | 1 | - | - | 1 |
| Necrosis, focal | - | - | - | - | - | - | - | - |
| Necrosis, focal with acute inflammation | - | - | - | - | - | - | - | - |
| Necrosis, periportal, acute | - | - | - | - | - | - | - | - |
| Inflammation, periportal, mixed cell, with oval cell hyperplasia | - | - | - | - | - | - | - | - |
| Necrosis, single cell | - | - | - | - | - | - | - | - |
| **Lung** |  |  |  |  |  |  |  |  |
| Athracosilicosis | - | - | 2 | - | - | - | - | 2 |
| Hemorrhage, focal | - | - | 2 | - | - | - | - | - |
