# Supplementary material for: Patterns of gene expression associated with recovery and injury in heat-stressed rats
Source: BMC Genomics. 2014 Dec 3;15(1):1058. doi: 10.1186/1471-2164-15-1058 (PMC4302131; doi:10.1186/1471-2164-15-1058)
Supplement: Supplementary file 3 — Additional file 3: Table S3: Table listing the histopathologies in animals 24 hours after heat stress. (DOCX 18 KB) [file 12864_2014_6768_MOESM3_ESM.docx]

**Additional File 3.** Histopathological findings in rats at 24 hours

|  | Animal Number | | | | | | | |
| --- | --- | --- | --- | --- | --- | --- | --- | --- |
|  | Heat-Stressed | | | | | | Control | |
|  | 24-7 | 24-8 | 24-9 | 24-10 | 24-11 | 24-12 | 24-1 | 24-2 |
| **Heart** |  |  |  |  |  |  |  |  |
| Cardiomyopathy | 1 | - | - | 0 | - | - | - | - |
| Inflammation, subacute, with cardiomyocyte degeneration and loss | - | - | - | 0 | - | - | - | - |
| **Kidney** |  |  |  |  |  |  |  |  |
| Chronic progressive nephropathy | - | - | - | - | - | - | - | - |
| Pelvic dilatation | - | - | - | - | - | - | - | - |
| Necrosis, tubular, diffuse, acute | - | - | - | - | 3 | - | - | - |
| Proteinosis | - | - | - | - | - | - | - | - |
| Proteinosis, tubular, diffuse | - | - | - | - | 3 | - | - | - |
| **Liver** |  |  |  |  |  |  |  |  |
| Inflammation, chronic | 1 | - | 1 | - | - | - | 1 | 1 |
| Pigment, intracellular | 1 | - | - | - | - | - | - | - |
| Necrosis, focal | - | - | - | - | - | - | - | 1 |
| Necrosis, focal with acute inflammation | - | - | - | - | - | - | - | - |
| Necrosis, periportal, acute | - | - | - | - | 4 | - | - | - |
| Inflammation, periportal, mixed cell, with oval cell hyperplasia | - | - | - | - | - | - | - | - |
| Necrosis, single cell | - | - | - | - | - | - | - | - |
| **Lung** |  |  |  |  |  |  |  |  |
| Athracosilicosis | 2 | - | - | - | - | - | - | - |
| Hemorrhage, focal | - | - | - | - | - | - | - | - |
