# Supplementary material for: Patterns of gene expression associated with recovery and injury in heat-stressed rats
Source: BMC Genomics. 2014 Dec 3;15(1):1058. doi: 10.1186/1471-2164-15-1058 (PMC4302131; doi:10.1186/1471-2164-15-1058)
Supplement: Supplementary file 4 — Additional file 4: Figure S1: Figure illustrating the histopathological evidence of kidney and liver injury that occurred in one rat 24 hours after heat stress. (DOCX 1 MB) [file 12864_2014_6768_MOESM4_ESM.docx]

**Additional File 4.** **Histopathological evidence of kidney and liver injury 24 hours after heat stress in a single rat (hematoxylin and eosin staining). (A)** 20x and **(B)** 200x magnification of kidney sections indicate degeneration and necrosis of the kidney tubular epithelium in a single heat-stressed rat at 24 hours. Pink fluid in the tubules (arrows) may indicate protein accumulation due to disruption of the functioning nephron. **(C)** 20x and **(D)** 200x magnification of liver sections from the same rat with focal areas of acute necrosis in hepatocytes surrounding the periportal regions of the liver (arrows).
