# Supplementary material for: Patterns of gene expression associated with recovery and injury in heat-stressed rats
Source: BMC Genomics. 2014 Dec 3;15(1):1058. doi: 10.1186/1471-2164-15-1058 (PMC4302131; doi:10.1186/1471-2164-15-1058)
Supplement: Supplementary file 10 — Additional file 10: Figure S2: Figure showing the self-organizing maps identifying gene nodes enriched in the liver at 24 hours. (DOCX 458 KB) [file 12864_2014_6768_MOESM10_ESM.docx]

**Additional File 10.** Liver self-organizing map (SOM). SOM clusters identified in the liver of heat-stressed animals (top row) and time-matched controls at 24 hours (n=6 rats per group). Node 1 identified 667 genes that are down-regulated and node 2 identified 670 genes up-regulated among the total background of 13787 genes in liver. The pathways enriched are below (**bolded terms** were also p<0.05, FDR-adjusted). Animal 12 showed evidence of histopathology in the liver (see Additional File 4).
